# Cut-off point, sensitivity and specificity for screening the reading fluency in children

**DOI:** 10.1590/2317-1782/20232021263en

**Published:** 2023-06-02

**Authors:** Hugo Cogo-Moreira, Giovanna Lima Molinari, Carolina Alves Ferreira de Carvalho, Adriana de Souza Batista Kida, Patrícia Silva Lúcio, Clara Regina Brandão de Avila

**Affiliations:** 1 Department of Education, ICT and Learning, Østfold University College - Fredrikstad, Norway.; 2 Departamento de Fonoaudiologia, Escola Paulista de Medicina, Universidade Federal de São Paulo - UNIFESP - São Paulo (SP), Brasil.; 3 Departamento de Psicologia e Psicanálise, Universidade Estadual de Londrina - UEL - Londrina (PR), Brasil.

**Keywords:** Reading, Statistics, Screening, Sensitivity and Specificity, Primary Education

## Abstract

**Purpose:**

to establish cut-off point for reading speed and accuracy, to obtain minimum values for comprehending texts, and allow classifying students from 2nd to 5th grade of elementary school according to good or poor reading performance.

**Methods:**

147 assessment protocols for oral reading and text comprehension of students from 3rd to 5th grade of Elementary School with and without reading difficulties were analyzed. The oral text reading rate and accuracy values were analyzed. ROC curves were constructed, and sensitivity and specificity calculated for each reading fluency parameter, and each school grade.

**Results:**

Sensitivity and specificity for measures of rate and accuracy in text reading were calculated for the 3rd, 4th, and 5th grades. Rate and precision under the ROC curve did not differ statistically. The values for the 2nd grade were mathematically estimated.

**Conclusion:**

The cutoff values expected for students from 2nd to 3rd grade were identified, with recommendations for using the oral text reading rate for reading comprehension screening procedures

## INTRODUCTION

During the learning of reading, the decoding conditions tend to change constantly, until the individual is able to read with the same speed and intonation traits that typifies their prosodic features of speech production during spontaneous oral speech, thus demonstrating that they achieved the automatic recognition of written words^(1,2)^, one of the components of reading fluency involved in literal comprehension^(3)^. Undoubtedly, the auditory language comprehension characteristics and some of its components, such as vocabulary, world knowledge, inference making ability and comprehension monitoring can be predictors of reading comprehension difficulties^(2)^.

Low fluency rates (correct number of words per minute) may interfere with reading comprehension^(4)^. Studies have indicated the relevance of measuring accuracy and considering the minimum proficiency levels as a condition to ensure literal comprehension and, therefore, to be able to appropriately evaluate reading skills^(2)^.

Fluent and accurate reading along with literal comprehension can be evaluated clinically as well as in the learning environment^(5)^. Furthermore, there is a decoding threshold established by investigative research, below which students could fail to understand written text^(6)^.

Similarly, researchers have also investigated to what extent increasing reading fluency rates can improve literal comprehension^(2)^. In this study, the author sought to identify the minimum values for speed and accuracy of oral reading rates required to allow the comprehension of the text, in the presence or absence of a reading disorder. The study showed an increase in reading rate values related to educational level, indicating improvement in literal comprehension only in the ranges from 35 correct number of words per minute (wpm) for students with reading disorders, to 75 wpm for neurotypical 2nd graders. For the 4th graders, the scores ranged from 40 wpm to 90 wpm, for the presence and absence of the disorder, respectively. In other words, reading with accuracy rates higher than these did not prove to be of any advantage for literal comprehension.

Brazilian researchers have published reference values for the average reading speed and accuracy of local schoolchildren, using different types of assessment - reading isolated words or text^(1,7)^ and procedures of the evaluation process^(8)^. However, none of these studies considered the minimum fluency rate needed for comprehension to occur, based on the above-mentioned rates.

In the context of school learning, comprehension monitoring strategies should be quickly implemented. They can be used to determine which students are at risk of presenting academic performance deficits. Such insufficiencies may appear either in the earliest stages of literacy development or in later phases where the literal comprehension demands tend to build up with each school year due to the increasing complexity of the texts^(1)^.In a learning environment, it can be an effective strategy for continuous monitoring of the student’s reading progress, allowing the identification of the condition based on two approaches: fail, if the student does not reach the cut-off value for average fluency standards expectations; and pass, if they perform equal to or higher than the cut-off for reading speed proficiency.

The present study was designed and executed to meet the needs of screening elementary school students for reading fluency to assess reading comprehension. To ensure that the decoding and fluency conditions are sufficient for the comprehension of the text, we conducted a retrospective research to investigate the reading rates and accuracy values of students with and without complaints of low reading proficiency, who participated in two other studies^(9,10)^.

This retrospective research aimed to establish a cut-off for reading rate and accuracy of students, from the 2nd to 5th grade of elementary school, aiming to classify readers according to the minimum reading fluency values required for comprehension, as well as to verify which of the two fluency parameters, whether rate or accuracy, is best suited to determine said reading condition. Hence, we proposed to establish cut-off values using screening tests for clinicians and researchers interested in studying the relationship between fluency and literal comprehension.

## METHODS

Retrospective exploratory study, developed from the data analysis of protocols standardized in previous research (Approved by the Research Ethics Committee of the Federal University of São Paulo, CEP-Unifesp/Number: 0839/06, 1490/08).

### Case study sample

We analyzed 147 reading assessment protocols of students enrolled in the 3rd up to 5th grades of public and private elementary schools in the city of São Paulo (SP). Notwithstanding the research objectives, the children were selected by their teachers. According to them, the students of the sample group had no complaints or indicators of motor or sensory deficits related to auditory or visual processing disorders (uncorrected); neurological, behavioral, or cognitive deficits or disorders; oral communication impairment; or indication of grade retention in school records. Then, the teachers classified the students based on their reading performance, thus creating two distinct groups: G1-school children with good reading performance (*n*=96); G2-school children with difficulties in reading fluency and/or text interpretation, classified as poor readers (*n*=51). This classification was used herein to conduct a dichotomous distribution of the children into two categories for further analysis with the ROC curve.

Children were also distributed by school year: 30 were enrolled in the 3rd grade (mean age=8.42; SD=0.05); 59 in the 4th grade (mean age=9.24; SD=0.22); and 58 in the 5th grade (mean age = 10.21; SD=0.07). Of the 147 children, 66.7% were girls. The parents or guardians signed the Informed Consent Form for the participation of their children in the research, providing the data required for the present study.

The available data referred only to these three school years. Consequently, the cut-off value to differentiate good and poor readers for the 2nd grade was statistically predicted. Although the evaluations were conducted over the course of 05 years, the procedures and protocols were standardized and applied on the second semester to all students.

### Data collection procedure

The oral reading of the text allowed the collection of data on reading rate and accuracy. A text was selected for each school year from the books used in Portuguese lessons (see Supplementary Material). The students were instructed to read the text aloud from the title. The individual readings were recorded for further analysis and calculation of two continuous measurements, namely, the rate (number of words read per minute, i.e., the total amount of words in the text divided by the reading time) and the accuracy (number of correctly read words per minute).

The correctness criterion was the automatic recognition of words, which implies that any reading repair or fix-up strategy was considered an error, regardless of whether to adjust the decoding or understanding of the text. For each assessment, comprehension was evaluated by reading 06 multiple-choice questions (see Supplementary Material) and by analyzing the oral retelling of the story, according to the number of ideas summarized by them^(9,10)^. The total score possibilities varied by text read: 27, 25 and 41 ideas for the texts provided for the 3rd, 4th, and 5th grades, respectively. The points scored in the retelling task were added to the scores of the answers to the questions correlated to the text.

### Statistical analysis procedure

To discriminate between good and poor understanders, the teachers were asked a simple question, which sought to infer whether the child in question had reading and/or literal comprehension difficulties or not: “Based on student X's performance in reading fluency and literal comprehension, would you classify them as a good or poor reader?”. From the categorization made by the teachers, ranking the student in one of these two possibilities, that is, good or poor readers/comprehenders, ROC (Receiver Operating Characteristics curve) curves were drafted and the areas below them were calculated. Using the teacher as a gold standard is a common procedure and it is usually done in research in various areas of child development studies^(11,12)^.

This method was used for the two outcome measures of the study: rate and accuracy. The continuous measurements that showed the best results would then be used to estimate the probable cut-off value for children in the 2nd year of schooling. If the measures did not differ, the most easily collected (i.e., the rate) would be used. The estimated cut-off for the 2nd graders was calculated with the progression of the cut-off for the children of the other school years via the *DataFit* 9 software (https://datafit.software.informer.com/9.0/). This way, the three cut-off values obtained, one for each school year, were plotted and, from them, different possibilities of growth trajectories were assessed against the addition of a new school year (i.e., the 2nd grade). Knowing that the distance between the four grades regarding the x-axis is equidistant, it was possible to estimate, for each predictive model evaluated, possibilities for the value referring to the y-axis (rate) for the 2nd grade. Non-linear trajectories were preferred, given the conformity of the three initial points.

## RESULTS

Table 1 shows the area under the ROC curve pertaining to rate and accuracy, their standard deviations and statistical significance, based on a non-parametric standard error assumption for the three studied years. From these areas, it was found that both measures presented values greater than 0.80. There is a non-linear progression of the cut-off values in relation to the child's development.

**Table 1:** Area under the ROC Curve for the three school years, cut-off values, sensitivity, and specificity for reading rate and accuracy measures

**Year**	**Measures**	**Area under the ROC Curve**	**S.D.**	**Cut-off**	**Sensitivity**	**Specificity**
**3rd**	**Rate**	0.970	0.027	65.670	0.900	0.100
	**Accuracy**	0.970	0.029	58.900	0.900	0.050
**4th**	**Rate**	0.866	0.055	70.930	0.857	0.132
	**Accuracy**	0.888	0.049	63.770	0.905	0.132
**5th**	**Rate**	0.849	0.051	91.375	0.900	0.289
	**Accuracy**	0.836	0.052	81.575	0.850	0.263

**Caption:** For all variables, p < 0.001. S.D; = *standard deviation*; **3rd grade:** Camargo^(13)^; **4th grade:** Lobato^(14)^; **5th grade:** Vasconcelos^(15)^.

**Figure 1:**
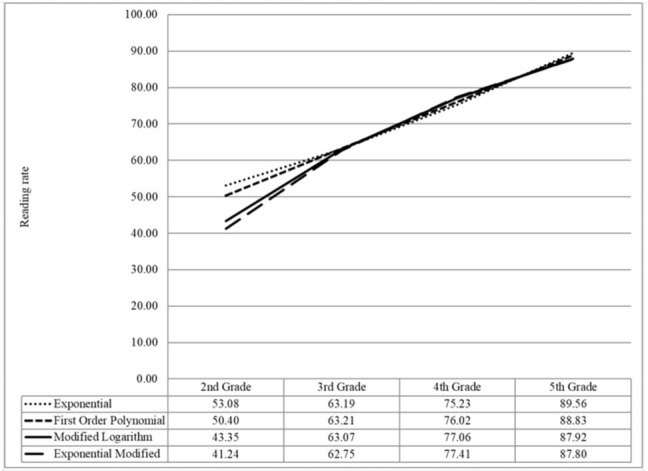
Cut-off values per year from the four estimation techniques

Statistically, the values of the area under the ROC curve did not differ from rate or accuracy (i.e., they produced similar areas under the curve for all years). Due to the efficiency of the rate data collection, this measure was used as estimation. Therefore, the cut-off estimation for the 2nd graders was calculated by four nonlinear functions: exponential, first order polynomial, modified logarithm, and modified exponential^(16)^. Since the results obtained with the ROC curve for each of the three years (the actual data), did not fit perfectly for all the non-linear functions tested, approximations were used. Originally, the values obtained for the 3rd, 4th and 5th graders are described in Table 1; however, Figure 1 shows that the original values are approximated to fit each of the four non-linear models used to estimate the value of the 2nd grade rate. Figure 1 depicts these approximations, and, for every real data, there is a function and an estimation of the specific cut-off value for the 2nd graders. An average of the three estimated cut-off values for the 2nd graders was plotted and this average refers to the final cut-off adopted for that year (i.e. (53.08 + 50.40 + 43.35 + 41.24)/4= 47.01).

## DISCUSSION

Screening procedures are efficient, quick to apply and are used for the necessary monitoring of reading development during school years^(1,5,8)^. The monitoring of reading skills can be performed not only to know the reading level of the student, but also for the early identification of reading difficulties to provide information for the necessary intervention and educational or clinical support^(1)^. This approach is usually accompanied by a Response to Intervention program along with support services for monitoring the student.

Knowing threshold values for reading fluency parameters can be useful to compose sample groups of students with the purpose of researching reading comprehension, since both competencies show association between different school years^(3,4).^The present study calculated and presented cut-off values for tracking by minimum rate or accuracy related to literal comprehension for the initial years of elementary school^(2,6)^. Considering that there were no apparent differences in the area under the ROC curve between reading rate and accuracy, it was decided to use a measure that can be applied easily and without further calculation, namely, the rate. Ergo, the wpm values of 65, 70 and 91 were considered minimum values for reading fluency as a condition to ensure literal comprehension in the 3rd, 4th, and 5th grades of elementary school, respectively. The texts used (see Supplementary Material) were a useful, quick, and easy tool to track speed associated with comprehension.

The values obtained for the rate and accuracy showed a decrease in specificity (i.e., probability of detection of true positive) from the 3rd to 5th grades (Table 1). It is possible that teachers can become less sensitive to the comprehension difficulties of older children. For example, a study with Brazilian schoolchildren^(17)^ found seven cases of poor reading of real words and 12 cases of decoding among 10-year-old children who were considered competent by the teachers. It is also possible that in this same group, on account of having a more developed reading fluency, other factors, such as vocabulary and monitoring capacity, may have interfered more with the literal comprehension.

One of the limitations of this study is that, although they showed significant differences in their performance, the sample groups were formed according to the teachers' opinion regarding the presence of generic reading difficulties or specific reading interpretation and literal comprehension^(7,10)^. The teacher’s reports and indications are seen as an important primary source of information for the other members of the educational team^(12,16)^ and, consequently, they play a fundamental role in monitoring the different stages of learning. However, other studies should be conducted with clinical groups with a specific learning disorder diagnosis, as to improve the information provided by the area under the ROC curve, especially for the final years in which the sensitivity and specificity values were less adequate, although still satisfactory (Table 1).

These values are expected to be similar, in case of any future research using a similar procedure, when using other narrative texts appropriate to the school year and with the same linguistic characteristics as those applied in this study. That is, research aiming to investigate if other texts would produce rates similar to the cut-off values obtained herein should still be carried out. It is known that texts may vary according to the type (narrative or expository), frequency of words, background knowledge necessary for understanding, and other aspects that tend to interfere with text complexity. Therefore, it is strongly recommended to use the same narrative texts applied in this study to identify the minimum fluency rate of correctly read words per minute to ensure literal comprehension. This recommendation also intends to reduce the potential margin of error.

Another important limitation is the fact that the cut-off values for younger children have been estimated (Figure 1). Nevertheless, it is necessary to recognize that estimated cut-off can produce reliable results in situations where measurement errors are acceptable, akin to the present study case^(18)^.

Still, it is emphasized that this estimated value is most suitable to scientific research, rather than to clinical or educational screening procedures. This limitation was overcome, to some extent, by estimating the cut-off values with the statistics used in the present study. Thus, clinicians and researchers can use an appropriate text for 2nd graders with the performance expectations for the adopted cut-off.

Researchers and clinicians will be free to benefit from the data for a more efficient assessment and future research, with a large enough sample of children in the studied age group providing cross-validation of the measures presented herein. Another possibility is to investigate whether the provided cut-off values are valid to classify school children as good or poor understanders, with independent samples as well, through cross-validation.

## CONCLUSION

Cut-off values were established for oral reading rate and accuracy for students from the 2nd to 5th grade, with the use of reading rate values for each school year being recommended for literacy screening procedures related to literal comprehension.

## Supplementary Material

Supplementary material accompanies this paper.

Texts selected for the research, extracted from books used in Portuguese school lessons, and correlated questions

This material is available as part of the online article from: https://doi.org/10.1590/2317-1782/20232021263en
