# Supplementary material for: Cut-off point, sensitivity and specificity for screening the reading fluency in children
Source: Codas. 2023 Jun 2;35(3):e20210263. doi: 10.1590/2317-1782/20232021263en (PMC10266799; doi:10.1590/2317-1782/20232021263en)
Supplement: Texts selected for the research, extracted from books used in Portuguese school lessons, and correlated questions [file codas-35-3-e20210263-suppl.pdf]

Material Suplementar

Textos selecionados para a pesquisa, extraídos de livros de Língua Portuguesa utilizados nas escolas, e questões correspondentes

O material escrito foi apresentado impresso em fonte Arial 12, com espaçamento 1,5. As leituras foram realizadas individualmente e gravadas para transcrição e posterior análise. Não foi estabelecido tempo limite e o acompanhamento com apoio digital durante a leitura não foi restringido.

Texto para 3ª série O VETERINÁRIO MALUCO

O veterinário maluco era o modo como o Doutor Inácio era conhecido na cidade. Ele não tinha nada de maluco. Era cientista e gostava muito de todos os animais.

Certa vez, o amestrador de pulgas de um circo veio procurá-lo:

- Doutor Inácio, a Margarida, a minha pulga mais inteligente, está doente. O senhor poderia curá-la?

- É claro que posso. Mas vai demorar um pouco. Preciso inventar um remédio para fazê-la crescer e outro para fazê-la voltar ao tamanho normal. Se não, não poderei examiná-la. Ela é muito pequena.

O Doutor Inácio foi para o seu laboratório e, duas horas depois, voltou com os remédios.

- Uma gotinha só é suficiente para a Margarida ficar do tamanho de um cachorro - disse o cientista.

A Margarida bebeu uma gotinha e cresceu. Então, o cientista a examinou.

- Com esse remedinho ela vai ficar curada. E ela saiu pulando pela casa, porque ficou curada. Então Doutor Inácio deu à pulga o remédio para diminuir e ela voltou ao seu tamanho normal.

Depois que o amestrador foi embora o cientista se lembrou que era hora de tomar o xarope para tosse.

Mas como era muito distraído, pegou o vidro errado.

- Que gigante é esse? - perguntou assustado o cientista. Meu Deus! Tomei o remédio errado! Estou do tamanho de uma pulga.

Camargo M. O veterinário maluco. Série Lagarta Pintada. 16ª ed. São Paulo: Ática; 1991

Texto para 4ª série A CORUJA E A ÁGUIA

Coruja e águia, depois de muita briga, resolveram fazer as pazes.

- Basta de guerra - disse a coruja. O mundo é tão grande, e tolice maior que o mundo é andarmos a comer os filhotes uma da outra.

- Perfeitamente - respondeu a águia. Também eu quero outra coisa.

- Nesse caso, combinemos isto: de ora em diante não comerás nunca os meus filhotes.

- Muito bem. Mas como posso distinguir os teus filhotes?

- Coisa fácil. Sempre que encontrares uns borrachos lindos, bem feitinhos de corpo, alegres, cheios de graça especial que não existe em filhotes de nenhuma outra ave, já sabes, são os meus.

- Está feito! - Concluiu a águia.

Dias depois, andando à caça, a águia encontrou um ninho com três monstrengos dentro, que piavam de bico muito aberto.

- Horríveis bichos! - disse ela. Vê-se logo que não são os filhos da coruja. E comeu-os.

Mas eram os filhotes da coruja. Ao regressar à toca, a triste mãe chorou amargamente o desastre e foi ajustar contas com a rainha das aves.

- Quê? - disse ela admirada. Eram teus filhos aqueles monstrinhos? Pois, olha, não se pareciam nada com o retrato que deles me fizeste...

Lobato M. (s/d) A coruja e a águia (https://contobrasileiro.com.br/a-coruja-e-a-aguia- fabula-de-monteiro-lobato/);

Texto para 5ª série **O PEQUENO ENGRAXATE**

Já passava de duas horas da tarde, gente andava pelas ruas e nada. Ninguém, nem para tirar o pó e soltar um tostão.

Fiquei perto de um poste de Rio - São Paulo e soltava de vez em quando minha voz fina:

- Graxa, freguês! Graxa, patrão! Graxa para ajudar o Natal do pobre!

Um carro de rico parou perto.

Eu aproveitei para gritar sem esperança alguma.

- Uma mãozinha doutor. Só para ajudar o Natal dos pobres!

A senhora bem vestida e os meninos atrás no carro ficaram me espiando, espiando. A senhora se comoveu.

- Coitadinho tão pequeno e tão pobrezinho! Dê qualquer coisa a ele, Arthur.

Mas o homem me analisou desconfiado.

- Isso é malandrinho e dos vivos. Ele está se aproveitando do tamanho e do dia.

Mesmo assim, eu vou dar. Vem cá menininho.

Abriu a bolsa e esticou a mão pela janela.

- Não senhora, obrigado. Eu não estou mentindo não. Só quem precisa muito trabalhar num dia de Natal.

Apanhei a caixa e coloquei no ombro e fui andando devagar. Não tinha nem força de tanta raiva.

Mas a porta do carro abriu-se e um menino desatou a correr para perto de mim.

- Tome garoto. Mamãe mandou dizer que ela não acredita que você seja mentiroso, não.

Botou mais cinco cruzeiros no meu bolso e nem esperou que eu agradecesse... só ouvi o ronco do motor se afastando.

Vasconcelos JM. (1975). *O Meu pé de laranja lima*. 2ª ed. São Paulo: Editora Melhoramentos, p.32-33

Textos utilizados para leitura silenciosa e análise da compreensão leitora

Para a avaliação da compreensão leitora, foi utilizado o protocolo de avaliação da compreensão da leitura[1]. Cada escolar foi instruído a ler silenciosamente um segundo texto, considerado adequado à sua idade e ano escolar, apresentado impresso em folha de papel sulfite com fonte Arial 11 e espaçamento duplo entre as linhas. Anteriormente à leitura, o escolar foi informado sobre a possibilidade de ler o texto mais de uma vez e sobre a não existência de tempo limite para realização da leitura. Também recebeu a instrução de que, imediatamente após a leitura, deveria recontar a história lida e em seguida, leria e responderia a seis questões de múltipla escolha referentes ao texto. O reconto foi feito oralmente e registrado em gravador digital para posterior transcrição canônica e análise.

Texto para 3ª série A APOSTA

Amélia é uma velhinha muito ativa e trabalhadeira. Um dia ela entrou no ônibus carregando uma cesta. O cobrador ouviu um barulho e perguntou-lhe:

- A senhora está levando uma galinha na cesta?

Amélia pensou, pensou e respondeu:

- Hum... Galinha? Não...Não há galinha nenhuma na cesta.

O cobrador insistiu tanto que Amélia resolveu fazer uma aposta:

---

[1] Carvalho CAF. Relação entre a função pragmática da linguagem e compreensão de textos. São Paulo. Dissertação [Mestrado] Universidade Federal de São Paulo; 2008.

- Senhor cobrador, se for galinha, eu desço agora do ônibus... Se não for, eu viajo de graça.

- Muito bem! - disse o cobrador confiante. Concordo!

Amélia, então, levantou a tampa da cesta e um galo de crista bem vermelhinha cantou satisfeito:

- Có-có-ró-có!

- Viu só? Eu não disse que não era galinha?!

O cobrador riu e deixou a velhinha viajar de graça.

Luciana M. M. Passos   Adaptação de conto popular http://profhelena4e5ano.blogspot.com/2011/08/aposta.html

Questões para avaliação da compreensão de leitura do texto "A Aposta"

1. O cobrador aceitou a aposta feita por Amélia porque:
a) ( ) queria ajudar a velhinha a viajar de graça
b) ( ) estava confiante de que ganharia a aposta
c) ( ) estava curioso para descobrir qual animal estava na cesta
d) ( ) estava em dúvida se Amélia carregava um animal na cesta

2. O barulho que o cobrador escutava era:
a) ( ) do cacarejar de um galo
b) ( ) do miado de um gato
c) ( ) do cacarejar de uma galinha
d) ( ) do ônibus

3. Amélia era:
a) ( ) uma velhinha muito bondosa
b) ( ) uma velhinha trabalhadeira
c) ( ) uma velhinha atrapalhada
d) ( ) uma velhinha curiosa

4. O cobrador perguntou a velhinha o que havia na cesta porque:
a) ( ) estava curioso
b) ( ) a cesta era grande e ele queria ajudá-la
c) ( ) estava irritado com o barulho
d) ( ) queria apenas começar uma conversa com a velhinha

5. Ao fazer a aposta, Amélia:
a) ( ) queria agradar o cobrador
b) ( ) queria aumentar a curiosidade do cobrador
c) ( ) queria descer logo do ônibus
d) ( ) queria tirar vantagem da situação

6. Quem ganhou a aposta?
a) ( ) a velhinha, pois na cesta havia uma galinha
b) ( ) o cobrador, pois conseguiu matar sua curiosidade
c) ( ) a velhinha, pois com sua esperteza conseguiu viajar de graça
d) ( ) o cobrador, pois tinha apostado que na cesta havia uma galinha

Texto para 4ª série **A COISA**

A casa do avô de Pedro era uma dessas casas antigas, grandes, que têm dois andares e mais um porão velho. Um dia Pedro resolveu ir lá embaixo procurar uns patins. Pegou uma lanterna e foi descendo as escadas com cuidado. No que foi, voltou aos berros:

— Fantasma! Uma coisa horrível! Um monstro com uma luz saindo da barriga.

Ninguém acreditou! Onde é que já se viu monstro com luz saindo da barriga? Então o vovô foi ver o que havia. E voltou correndo como o Pedro:

— A Coisa! — ele gritava — A Coisa! É muito alta, com os olhos brilhantes, como se fossem de vidro! E na cabeça uns tufos espetados para todos os lados!

Dona Julinha, a avó do Pedro, era a única que não estava impressionada. Então ela foi ver o que estava acontecendo. Foi descendo as escadas devagar, abrindo as janelas que encontrava. A família veio atrás toda assustada, morrendo de medo do monstro, fantasma, fosse lá o que fosse.

Até que chegaram lá embaixo e Dona Julinha abriu a última janela. Então todos começaram a rir, muito envergonhados. A Coisa era... um espelho! Cada um que descia as escadas, no escuro, via uma coisa diferente no espelho. E todos eles pensavam que tinham visto... a Coisa.

Adaptado de Ruth Rocha

Referência: Salles JF, Parente MAMPP. 2004. Compreensão textual em alunos de segunda e terceira séries: uma abordagem cognitiva. *Estudos de Psicologia*, 9(1): 71-80

Questões para avaliação da compreensão de leitura do texto "A coisa"

1. O que Pedro estava procurando no porão?
a) ( ) uma lanterna
b) ( ) uns brinquedos
c) ( ) um espelho
d) ( ) uns patins

2. Como era a casa dos avós de Pedro?
a) ( ) pequena e nova
b) ( ) de madeira
c) ( ) grande e antiga
d) ( ) branca e com janelas grandes

3. Quem é que esclareceu o mistério da Coisa?

a) ( ) o avô de Pedro
b) ( ) a avó de Pedro
c) ( ) o próprio Pedro
d) ( ) o tio de Pedro

4. Por que Pedro pegou uma lanterna para ir até o porão?
a) ( ) porque ele não queria que ninguém o incomodasse ao brincar com a lanterna.
b) ( ) porque lá embaixo estava muito escuro.
c) ( ) porque o porão é o lugar de guardar a lanterna.
d) ( ) porque sua avó mandou-o trocar as lâmpadas do porão.

5. Por que o avô de Pedro via uma coisa com olhos brilhantes, como se fossem de vidro?
a) ( ) porque ele usava óculos, que refletiram no espelho parecendo um monstro.
b) ( ) porque ele estava sonhando.
c) ( ) porque lá embaixo havia vidros quebrados.
d) ( ) porque a coisa tinha olhos muito grandes.

6. Por que cada um que descia a escada via uma coisa diferente?
a) ( ) porque havia vários fantasmas lá embaixo.
b) ( ) porque a Coisa se escondia atrás dos móveis do porão.
c) ( ) porque o que cada um via era sua própria imagem refletida no espelho.
d) ( ) porque todos eles estavam sonhando.

Texto para 5ª série **O TELEGRAMA**

Toca a campainha.

- Aqui é o 934?

- Sim.

- Por favor, assine aqui.

O carteiro entrega o telegrama ao morador. Quando se vira para ir embora, o morador grita por ele.

- Ei, espera aí um pouquinho! O senhor se enganou, este telegrama não é para mim.

- Como não é?

- Ora, e desde quando eu me chamo Filomena?

- Sei lá, talvez sua esposa, filha ou coisa parecida.

- Não senhor, eu moro sozinho.

- Mas não é aqui o 934?

- Já te disse que é.

- Então pronto, ora bolas. Se aí está escrito 934 e se aqui é o 934, então não tem nada de errado. É aqui e pronto.

- Mas houve um engano. Eu não posso ficar com uma correspondência que não me pertence.

- E que é que eu posso fazer? Meu trabalho é esse. Eu não posso entregar um telegrama no 935 se é no 934, ou posso?

- Não, não pode. Mas se o senhor o devolver para o correio, está resolvido. Eu é que não tenho nada a ver com isso!

- Como não? O senhor não mora no 934?

- Moro.

- O telegrama não é para o 934? Então o senhor vai ter que ficar com isso. Que culpa tenho eu se não mora nenhuma Filomena aqui?

- E se for algo importante? Alguma coisa urgente?

- O senhor se vira, eu só cumpri meu trabalho.

- Então eu vou abrir.

- Ah! Mas isso é crime! Violação de correspondência!

- Como crime? O telegrama não é para o 934?

- É, uai!

- É onde é o 934?

- É aqui, uai!

- Então pronto. O senhor mesmo não ta querendo que eu fique com ele?

- É, nesse ponto o senhor tem razão. Então vamos ler o que está escrito aí.

O morador lê em voz alta:

- "Querida sobrinha, mando dinheiro herança vovô".

E com o rosto triste continuou:

- Puxa vida, o vovô morreu!

- Vovô? Mas como? Que negócio é esse? - disse o carteiro sem nada entender.

- Ora, rapaz, numa hora dessas o senhor me vem com perguntas cretinas! Não respeita o sofrimento dos outros? Passar bem!

O morador entra na casa, falando em voz alta:

- Pobre vovô!

Autor: Alexandre Azevedo

https://www.wattpad.com/191686673-crônicas-para-ler-e-reler-o-telegrama

Questões para avaliação da compreensão de leitura do texto "O telegrama"

1. Em que local se passa a história?
a) ( ) No correio

b) ( ) Na casa do vovô
c) ( ) Na casa de número 935
d) ( ) Na casa de número 934

2. Por que o morador recusou-se a receber o telegrama?
a) ( ) Porque havia brigado com Filomena e não queria ter notícias dela
b) ( ) Porque o telegrama era para sua esposa que não estava em casa
c) ( ) Porque o telegrama estava endereçado para outra pessoa
d) ( ) Porque não queria assinar a documentação do correio

3. O telegrama informava, sobre uma herança, para:
a) ( ) Filomena.
b) ( ) o morador.
c) ( ) a esposa do morador.
d) ( ) a sobrinha do morador.

4. Quando o carteiro diz: "Então o senhor vai ter que ficar com isso. Que culpa tenho eu se não mora nenhuma Filomena aqui?", ele estava querendo:
a) ( ) desculpar-se com o morador pelo equívoco
b) ( ) propor uma solução justa para o impasse
c) ( ) convencer o morador de que Filomena mora no 934
d) ( ) livrar-se do problema

5. Quem enviou o telegrama que informava sobre a herança?
a) ( ) o avô do morador
b) ( ) a sobrinha do morador
c) ( ) a sobrinha de Filomena
d) ( ) a tia de Filomena

6. O morador, finalmente, aceitou o telegrama porque:
a) ( ) Preocupou-se, pois a mensagem poderia ser urgente
b) ( ) Estava interessado na herança
c) ( ) Lembrou-se que Filomena era sua prima e decidiu entregar-lhe o telegrama
d) ( ) Gostava muito do avô e ficou triste com a notícia de sua morte

Material Suplementar

<u>Textos selecionados para a pesquisa, extraídos de livros de Língua Portuguesa utilizados nas escolas, e questões correspondentes</u>

O material escrito foi apresentado impresso em fonte Arial 12, com espaçamento 1,5. As leituras foram realizadas individualmente e gravadas para transcrição e posterior análise. Não foi estabelecido tempo limite e o acompanhamento com apoio digital durante a leitura não foi restringido.

Texto para 3ª série **O VETERINÁRIO MALUCO**

O veterinário maluco era o modo como o Doutor Inácio era conhecido na cidade. Ele não tinha nada de maluco. Era cientista e gostava muito de todos os animais.

Certa vez, o amestrador de pulgas de um circo veio procurá-lo:

- Doutor Inácio, a Margarida, a minha pulga mais inteligente, está doente. O senhor poderia curá-la?

- É claro que posso. Mas vai demorar um pouco. Preciso inventar um remédio para fazê-la crescer e outro para fazê-la voltar ao tamanho normal. Se não, não poderei examiná-la. Ela é muito pequena.

O Doutor Inácio foi para o seu laboratório e, duas horas depois, voltou com os remédios.

- Uma gotinha só é suficiente para a Margarida ficar do tamanho de um cachorro - disse o cientista.

A Margarida bebeu uma gotinha e cresceu. Então, o cientista a examinou.

- Com esse remedinho ela vai ficar curada. E ela saiu pulando pela casa, porque ficou curada. Então Doutor Inácio deu à pulga o remédio para diminuir e ela voltou ao seu tamanho normal.

Depois que o amestrador foi embora o cientista se lembrou que era hora de tomar o xarope para tosse.

Mas como era muito distraído, pegou o vidro errado.

- Que gigante é esse? - perguntou assustado o cientista. Meu Deus! Tomei o remédio errado! Estou do tamanho de uma pulga.

Camargo M. O veterinário maluco. Série Lagarta Pintada. 16ª ed. São Paulo: Ática; 1991

Texto para 4ª série **A CORUJA E A ÁGUIA**

Coruja e águia, depois de muita briga, resolveram fazer as pazes.

- Basta de guerra - disse a coruja. O mundo é tão grande, e tolice maior que o mundo é andarmos a comer os filhotes uma da outra.

- Perfeitamente - respondeu a águia. Também eu quero outra coisa.

- Nesse caso, combinemos isto: de ora em diante não comerás nunca os meus filhotes.

- Muito bem. Mas como posso distinguir os teus filhotes?

- Coisa fácil. Sempre que encontrares uns borrachos lindos, bem feitinhos de corpo, alegres, cheios de graça especial que não existe em filhotes de nenhuma outra ave, já sabes, são os meus.

- Está feito! - Concluiu a águia.

Dias depois, andando à caça, a águia encontrou um ninho com três monstrengos dentro, que piavam de bico muito aberto.

- Horríveis bichos! - disse ela. Vê-se logo que não são os filhos da coruja. E comeu-os.

Mas eram os filhotes da coruja. Ao regressar à toca, a triste mãe chorou amargamente o desastre e foi ajustar contas com a rainha das aves.

- Quê? - disse ela admirada. Eram teus filhos aqueles monstrinhos? Pois, olha, não se pareciam nada com o retrato que deles me fizeste...

Lobato M. (s/d) A coruja e a águia (https://contobrasileiro.com.br/a-coruja-e-a-aguia- fabula-de-monteiro-lobato/);

Texto para 5ª série **O PEQUENO ENGRAXATE**

Já passava de duas horas da tarde, gente andava pelas ruas e nada. Ninguém, nem para tirar o pó e soltar um tostão.

Fiquei perto de um poste de Rio - São Paulo e soltava de vez em quando minha voz fina:

- Graxa, freguês! Graxa, patrão! Graxa para ajudar o Natal do pobre!

Um carro de rico parou perto.

Eu aproveitei para gritar sem esperança alguma.

- Uma mãozinha doutor. Só para ajudar o Natal dos pobres!

A senhora bem vestida e os meninos atrás no carro ficaram me espiando, espiando. A senhora se comoveu.

- Coitadinho tão pequeno e tão pobrezinho! Dê qualquer coisa a ele, Arthur.

Mas o homem me analisou desconfiado.

- Isso é malandrinho e dos vivos. Ele está se aproveitando do tamanho e do dia.

Mesmo assim, eu vou dar. Vem cá menininho.

Abriu a bolsa e esticou a mão pela janela.

- Não senhora, obrigado. Eu não estou mentindo não. Só quem precisa muito trabalhar num dia de Natal.

Apanhei a caixa e coloquei no ombro e fui andando devagar. Não tinha nem força de tanta raiva.

Mas a porta do carro abriu-se e um menino desatou a correr para perto de mim.

- Tome garoto. Mamãe mandou dizer que ela não acredita que você seja mentiroso, não.

Botou mais cinco cruzeiros no meu bolso e nem esperou que eu agradecesse... só ouvi o ronco do motor se afastando.

Vasconcelos JM. (1975). *O Meu pé de laranja lima*. 2ª ed. São Paulo: Editora Melhoramentos, p.32-33

Textos utilizados para leitura silenciosa e análise da compreensão leitora

Para a avaliação da compreensão leitora, foi utilizado o protocolo de avaliação da compreensão da leitura[1]. Cada escolar foi instruído a ler silenciosamente um segundo texto, considerado adequado à sua idade e ano escolar, apresentado impresso em folha de papel sulfite com fonte Arial 11 e espaçamento duplo entre as linhas. Anteriormente à leitura, o escolar foi informado sobre a possibilidade de ler o texto mais de uma vez e sobre a não existência de tempo limite para realização da leitura. Também recebeu a instrução de que, imediatamente após a leitura, deveria recontar a história lida e em seguida, leria e responderia a seis questões de múltipla escolha referentes ao texto. O reconto foi feito oralmente e registrado em gravador digital para posterior transcrição canônica e análise.

Texto para 3ª série A APOSTA

Amélia é uma velhinha muito ativa e trabalhadeira. Um dia ela entrou no ônibus carregando uma cesta. O cobrador ouviu um barulho e perguntou-lhe:

- A senhora está levando uma galinha na cesta?

Amélia pensou, pensou e respondeu:

- Hum... Galinha? Não...Não há galinha nenhuma na cesta.

O cobrador insistiu tanto que Amélia resolveu fazer uma aposta:

[1] Carvalho CAF. Relação entre a função pragmática da linguagem e compreensão de textos. São Paulo. Dissertação [Mestrado] Universidade Federal de São Paulo; 2008.

- Senhor cobrador, se for galinha, eu desço agora do ônibus... Se não for, eu viajo de graça.

- Muito bem! - disse o cobrador confiante. Concordo!

Amélia, então, levantou a tampa da cesta e um galo de crista bem vermelhinha cantou satisfeito:

- Có-có-ró-có!

- Viu só? Eu não disse que não era galinha?!

O cobrador riu e deixou a velhinha viajar de graça.

Luciana M. M. Passos Adaptação de conto popular http://profhelena4e5ano.blogspot.com/2011/08/aposta.html

Questões para avaliação da compreensão de leitura do texto "A Aposta"

1. O cobrador aceitou a aposta feita por Amélia porque:
a) ( ) queria ajudar a velhinha a viajar de graça
b) ( ) estava confiante de que ganharia a aposta
c) ( ) estava curioso para descobrir qual animal estava na cesta
d) ( ) estava em dúvida se Amélia carregava um animal na cesta

2. O barulho que o cobrador escutava era:
a) ( ) do cacarejar de um galo
b) ( ) do miado de um gato
c) ( ) do cacarejar de uma galinha
d) ( ) do ônibus

3. Amélia era:
a) ( ) uma velhinha muito bondosa
b) ( ) uma velhinha trabalhadeira
c) ( ) uma velhinha atrapalhada
d) ( ) uma velhinha curiosa

4. O cobrador perguntou a velhinha o que havia na cesta porque:
a) ( ) estava curioso
b) ( ) a cesta era grande e ele queria ajudá-la
c) ( ) estava irritado com o barulho
d) ( ) queria apenas começar uma conversa com a velhinha

5. Ao fazer a aposta, Amélia:
a) ( ) queria agradar o cobrador
b) ( ) queria aumentar a curiosidade do cobrador
c) ( ) queria descer logo do ônibus
d) ( ) queria tirar vantagem da situação

6. Quem ganhou a aposta?
a) ( ) a velhinha, pois na cesta havia uma galinha
b) ( ) o cobrador, pois conseguiu matar sua curiosidade
c) ( ) a velhinha, pois com sua esperteza conseguiu viajar de graça
d) ( ) o cobrador, pois tinha apostado que na cesta havia uma galinha

Texto para 4ª série **A COISA**

A casa do avô de Pedro era uma dessas casas antigas, grandes, que têm dois andares e mais um porão velho. Um dia Pedro resolveu ir lá embaixo procurar uns patins. Pegou uma lanterna e foi descendo as escadas com cuidado. No que foi, voltou aos berros:

— Fantasma! Uma coisa horrível! Um monstro com uma luz saindo da barriga.

Ninguém acreditou! Onde é que já se viu monstro com luz saindo da barriga? Então o vovô foi ver o que havia. E voltou correndo como o Pedro:

— A Coisa! — ele gritava — A Coisa! É muito alta, com os olhos brilhantes, como se fossem de vidro! E na cabeça uns tufos espetados para todos os lados!

Dona Julinha, a avó do Pedro, era a única que não estava impressionada. Então ela foi ver o que estava acontecendo. Foi descendo as escadas devagar, abrindo as janelas que encontrava. A família veio atrás toda assustada, morrendo de medo do monstro, fantasma, fosse lá o que fosse.

Até que chegaram lá embaixo e Dona Julinha abriu a última janela. Então todos começaram a rir, muito envergonhados. A Coisa era... um espelho! Cada um que descia as escadas, no escuro, via uma coisa diferente no espelho. E todos eles pensavam que tinham visto... a Coisa.

Adaptado de Ruth Rocha

Referência: Salles JF, Parente MAMPP. 2004. Compreensão textual em alunos de segunda e terceira séries: uma abordagem cognitiva. *Estudos de Psicologia*, 9(1): 71-80

Questões para avaliação da compreensão de leitura do texto "A coisa"

1. O que Pedro estava procurando no porão?
a) ( ) uma lanterna
b) ( ) uns brinquedos
c) ( ) um espelho
d) ( ) uns patins

2. Como era a casa dos avós de Pedro?
a) ( ) pequena e nova
b) ( ) de madeira
c) ( ) grande e antiga
d) ( ) branca e com janelas grandes

3. Quem é que esclareceu o mistério da Coisa?

a) ( ) o avô de Pedro
b) ( ) a avó de Pedro
c) ( ) o próprio Pedro
d) ( ) o tio de Pedro

4. Por que Pedro pegou uma lanterna para ir até o porão?
a) ( ) porque ele não queria que ninguém o incomodasse ao brincar com a lanterna.
b) ( ) porque lá embaixo estava muito escuro.
c) ( ) porque o porão é o lugar de guardar a lanterna.
d) ( ) porque sua avó mandou-o trocar as lâmpadas do porão.

5. Por que o avô de Pedro via uma coisa com olhos brilhantes, como se fossem de vidro?
a) ( ) porque ele usava óculos, que refletiram no espelho parecendo um monstro.
b) ( ) porque ele estava sonhando.
c) ( ) porque lá embaixo havia vidros quebrados.
d) ( ) porque a coisa tinha olhos muito grandes.

6. Por que cada um que descia a escada via uma coisa diferente?
a) ( ) porque havia vários fantasmas lá embaixo.
b) ( ) porque a Coisa se escondia atrás dos móveis do porão.
c) ( ) porque o que cada um via era sua própria imagem refletida no espelho.
d) ( ) porque todos eles estavam sonhando.

Texto para 5ª série **O TELEGRAMA**

Toca a campainha.

- Aqui é o 934?

- Sim.

- Por favor, assine aqui.

O carteiro entrega o telegrama ao morador. Quando se vira para ir embora, o morador grita por ele.

- Ei, espera aí um pouquinho! O senhor se enganou, este telegrama não é para mim.

- Como não é?

- Ora, e desde quando eu me chamo Filomena?

- Sei lá, talvez sua esposa, filha ou coisa parecida.

- Não senhor, eu moro sozinho.

- Mas não é aqui o 934?

- Já te disse que é.

- Então pronto, ora bolas. Se aí está escrito 934 e se aqui é o 934, então não tem nada de errado. É aqui e pronto.

- Mas houve um engano. Eu não posso ficar com uma correspondência que não me pertence.

- E que é que eu posso fazer? Meu trabalho é esse. Eu não posso entregar um telegrama no 935 se é no 934, ou posso?

- Não, não pode. Mas se o senhor o devolver para o correio, está resolvido. Eu é que não tenho nada a ver com isso!

- Como não? O senhor não mora no 934?

- Moro.

- O telegrama não é para o 934? Então o senhor vai ter que ficar com isso. Que culpa tenho eu se não mora nenhuma Filomena aqui?

- E se for algo importante? Alguma coisa urgente?

- O senhor se vira, eu só cumpri meu trabalho.

- Então eu vou abrir.

- Ah! Mas isso é crime! Violação de correspondência!

- Como crime? O telegrama não é para o 934?

- É, uai!

- É onde é o 934?

- É aqui, uai!

- Então pronto. O senhor mesmo não ta querendo que eu fique com ele?

- É, nesse ponto o senhor tem razão. Então vamos ler o que está escrito aí.

O morador lê em voz alta:

- "Querida sobrinha, mando dinheiro herança vovô".

E com o rosto triste continuou:

- Puxa vida, o vovô morreu!

- Vovô? Mas como? Que negócio é esse? - disse o carteiro sem nada entender.

- Ora, rapaz, numa hora dessas o senhor me vem com perguntas cretinas! Não respeita o sofrimento dos outros? Passar bem!

O morador entra na casa, falando em voz alta:

- Pobre vovô!

Autor: Alexandre Azevedo

https://www.wattpad.com/191686673-crônicas-para-ler-e-reler-o-telegrama

Questões para avaliação da compreensão de leitura do texto "O telegrama"

1. Em que local se passa a história?
a) ( ) No correio

b) ( ) Na casa do vovô
c) ( ) Na casa de número 935
d) ( ) Na casa de número 934

2. Por que o morador recusou-se a receber o telegrama?
a) ( ) Porque havia brigado com Filomena e não queria ter notícias dela
b) ( ) Porque o telegrama era para sua esposa que não estava em casa
c) ( ) Porque o telegrama estava endereçado para outra pessoa
d) ( ) Porque não queria assinar a documentação do correio

3. O telegrama informava, sobre uma herança, para:
a) ( ) Filomena.
b) ( ) o morador.
c) ( ) a esposa do morador.
d) ( ) a sobrinha do morador.

4. Quando o carteiro diz: “Então o senhor vai ter que ficar com isso. Que culpa tenho eu se não mora nenhuma Filomena aqui?”, ele estava querendo:
a) ( ) desculpar-se com o morador pelo equívoco
b) ( ) propor uma solução justa para o impasse
c) ( ) convencer o morador de que Filomena mora no 934
d) ( ) livrar-se do problema

5. Quem enviou o telegrama que informava sobre a herança?
a) ( ) o avô do morador
b) ( ) a sobrinha do morador
c) ( ) a sobrinha de Filomena
d) ( ) a tia de Filomena

6. O morador, finalmente, aceitou o telegrama porque:
a) ( ) Preocupou-se, pois a mensagem poderia ser urgente
b) ( ) Estava interessado na herança
c) ( ) Lembrou-se que Filomena era sua prima e decidiu entregar-lhe o telegrama
d) ( ) Gostava muito do avô e ficou triste com a notícia de sua morte
