# Supplementary material for: Cut-off point, sensitivity and specificity for screening the reading fluency in children
Source: Codas. 2023 Jun 2;35(3):e20210263. doi: 10.1590/2317-1782/20232021263en (PMC10266799; doi:10.1590/2317-1782/20232021263en)
Supplement: Translation

# Translation: Pontos de corte, sensibilidade e especificidade para rastreamento da fluência leitora em crianças

**DOI:** 10.1590/2317-1782/20232021263pt

**Authors:** Hugo Cogo-Moreira, Giovanna Lima Molinari, Carolina Alves Ferreira de Carvalho, Adriana de Souza Batista Kida, Patrícia Silva Lúcio, Clara Regina Brandão de Avila

**Affiliations:** 1 Department of Education, ICT and Learning, Østfold University College - Fredrikstad, Norway.; 2 Departamento de Fonoaudiologia, Escola Paulista de Medicina, Universidade Federal de São Paulo - UNIFESP - São Paulo (SP), Brasil.; 3 Departamento de Psicologia e Psicanálise, Universidade Estadual de Londrina - UEL - Londrina (PR), Brasil.

**Keywords:** Leitura, Rastreamento, Estatística, Sensibilidade e Especificidade, Ensino Fundamental

## Abstract

**Objetivo:**

estabelecer pontos de corte para valores de velocidade e acurácia de leitura, a fim de obter valores mínimos para compreensão de textos, e permitir classificar alunos de 2º a 5º ano do Ensino Fundamental, de acordo com bom ou pobre desempenho em leitura.

**Método:**

foram analisados ​​147 protocolos de avaliação de leitura oral e compreensão textual de escolares de 3º a 5º ano com e sem dificuldades de leitura. A taxa de leitura oral de texto e os valores de acurácia foram analisados. Curvas ROC foram construídas e a sensibilidade e a especificidade, calculadas para cada parâmetro de fluência de leitura por ano escolar.

**Resultados:**

A sensibilidade e a especificidade para medidas de taxa e acurácia na leitura de texto foram calculadas para o 3º, 4º e 5º anos. A taxa e a precisão sob a curva ROC não diferiram estatisticamente. Os valores do 2º ano foram estimados matematicamente.

**Conclusão:**

Foram identificados os valores de corte esperados para escolares do 2º ao 5º ano, com recomendações de utilização da taxa de leitura de texto oral para procedimentos de rastreamento da compreensão leitora.

## INTRODUÇÃO

Durante o aprendizado da leitura, as condições de decodificação modificam-se constantemente, até que o leitor demonstre ao ler, a mesma velocidade e entonação que imprime em sua produção de fala durante o discurso oral espontâneo, mostrando, assim, que alcançou o reconhecimento automático das palavras escritas^(1,2)^, um dos componentes da fluência de leitura envolvidos na compreensão do texto lido^(3)^. Indubitavelmente, características da compreensão linguística auditiva e de alguns de seus componentes como o vocabulário, o conhecimento de mundo, a capacidade de elaborar inferências e a de monitorar o próprio entendimento, podem predizer dificuldades de compreensão de leitura^(2)^.

Baixas taxas de acurácia de leitura (número de palavras lidas corretamente por minuto) podem interferir na compreensão do texto^(4)^. Algumas pesquisas têm demonstrado a pertinência de buscar e considerar valores mínimos de acurácia como condição para garantir que a compreensão leitora possa acontecer e, portanto, ser avaliada de forma apropriada^(2)^.

A leitura fluente e acurada e a compreensão do texto lido podem ser avaliadas clinicamente e no ambiente escolar^(5)^. Pesquisadores investigaram um valor limite de decodificação abaixo do qual estariam estudantes que podem falhar em compreender textos^(6)^.

Com propósito semelhante, investigou-se até que ponto aumentar a taxa de leitura pode melhorar a compreensão do texto lido^(2)^. Nesse estudo, a autora procurou identificar quais taxas de leitura oral poderiam ser o valor mínimo de velocidade e precisão para permitir compreensão do texto, na presença ou ausência de transtorno de leitura. A pesquisa mostrou aumento dos valores de taxa de leitura em função da escolaridade, com melhora da compreensão leitora apenas nas faixas entre 35 palavras lidas corretamente por minuto (p.c.p.m.) para o transtorno de leitura e 75 p.c.p.m. para escolares típicos no 2º ano. Para os de 4º ano, entre 40 p.c.p.m. e 90 p.c.p.m., respectivamente à presença, ou não, do transtorno. Ou seja, ler com taxas superiores a essas não se revelou nenhuma vantagem para a compreensão de leitura.

Estudos nacionais publicaram valores de referência para médias de velocidade e acurácia de leitura de escolares brasileiros, nas suas diferentes formas de avaliação - com leitura de palavras isoladas ou de texto^(1,7)^ e procedimentos seguidos nessas avaliações^(8)^. Entretanto, nenhum desses estudos considerou o desempenho mínimo em fluência para o alcance da compreensão, a partir das taxas mencionadas.

No âmbito da aprendizagem escolar, procedimentos de rastreamento das condições de leitura devem ser de rápida aplicação. São utilizados para a identificação de escolares em risco de apresentarem déficits de desempenho acadêmico. Tais déficits podem aparecer desde etapas mais remotas do desenvolvimento relacionadas à época da alfabetização até fases ulteriores de demandas de compreensão leitora de textos cujas complexidades aumentam a cada ano escolar^(1)^. Em ambiente educacional, pode ser um procedimento eficaz de monitoramento continuado do progresso da leitura, possibilitando a identificação da condição através de duas categorias: falha, se não atingir o ponto de corte; e passa, caso evidencie desempenho igual ou superior ao ponto de corte.

Este estudo foi elaborado e executado para atender às necessidades de triar estudantes do Ensino Fundamental I pela fluência de leitura com vistas à avaliação da compreensão leitora. Para garantir condições de decodificação e fluência, suficientes para a compreensão do texto investigaram-se, retrospectivamente, os valores de taxa e acurácia de leitura textual de escolares com e sem queixa de problemas de leitura, que participaram de outros dois estudos^(9,10)^^.^

O objetivo desta pesquisa retrospectiva foi estabelecer pontos de corte de taxa e acurácia na leitura de escolares, do 2^º^ ao 5^º^ ano do Ensino Fundamental, a fim de separar leitores por valores mínimos de fluência de leitura para a compreensão e verificar qual dos dois parâmetros de fluência, se taxa ou acurácia, melhor determina essa condição de leitura. Desse modo, propôs-se estabelecer pontos de corte por meio de uso de tarefas de rastreamento para o clínico e o pesquisador interessados em estudar a relação entre fluência e compreensão de leitura.

## MÉTODO

Estudo retrospectivo, de caráter exploratório, realizado a partir da análise de dados de protocolos constituídos em pesquisas anteriores (CEP-Unifesp/Aprovações: 0839/06, 1490/08).

### Amostra

Analisaram-se 147 protocolos de avaliação da leitura de escolares matriculados do 3º ao 5º ano do Ensino Fundamental de escolas públicas e particulares do município de São Paulo (SP). Independentemente dos objetivos das pesquisas, as crianças foram selecionadas por seus professores. Segundo eles, estavam ausentes queixas ou indicadores de: déficits motores ou sensoriais auditivos ou visuais (não corrigidos); déficits ou distúrbios neurológicos, comportamentais ou cognitivos; alterações da comunicação oral; indicação de retenção no histórico escolar. A seguir, os professores separaram os escolares segundo julgaram seus desempenhos em leitura e, assim, definiram-se dois grupos distintos: G1-escolares com bom desempenho em leitura (n=96); G2-escolares com dificuldades de fluência leitora e/ou de interpretação de texto, classificados como maus leitores (n=51). Essa classificação foi utilizada, no presente estudo, para distribuir as crianças dicotomicamente em duas categorias para posterior análise nas curvas ROC.

As crianças também foram distribuídas por ano escolar: 30 eram do 3º ano (média de idade=8,42; DP=0,05); 59 do 4º ano (média de idade=9,24; DP=0,22); e 58 do 5º ano (média de idade = 10,21; DP=0,07). Das 147 crianças, 66,7% eram meninas. Os pais ou responsáveis assinaram o Termo de Consentimento Livre e Esclarecido para participação de seus filhos e filhas nas pesquisas, cujos dados permitiram este estudo.

Os únicos dados disponíveis eram dos três anos escolares. Assim, o ponto de corte para diferenciar bons e maus leitores para o 2º ano foi predito estatisticamente. Embora as avaliações tenham sido realizadas ao longo de 05 anos, os procedimentos e protocolos foram idênticos e aplicados com todos os escolares no segundo semestre letivo.

### Procedimento de coleta dos dados

A leitura oral de texto permitiu coletar informações sobre taxa e acurácia leitora. Um texto para cada ano escolar foi selecionado dos livros de Língua Portuguesa utilizados nas escolas (Material Suplementar). Os escolares foram instruídos a ler o texto em voz alta a partir do título. As leituras, individuais, foram gravadas para posterior análise e cálculo de duas medidas contínuas, a saber, a taxa (número de palavras lidas por minuto, i.e., total de palavras no texto dividido pelo tempo de leitura) e a acurácia (número de palavras corretas lidas por minuto).

O critério de acerto foi o de reconhecimento automático das palavras, o que implica dizer que qualquer atividade de reparo da leitura foi considerada erro, não importando se para ajuste da decodificação ou compreensão. Para cada texto, após a leitura, a compreensão foi avaliada por 06 questões de múltipla escolha (Material Suplementar) e pela análise do reconto oral da história lida, segundo o número de ideias apresentadas^(9,10)^. A possibilidade do total de pontos variou por texto lido: 27, 25 e 41 ideias para os textos do 3^o^, 4^o^ e 5^o^ ano, respectivamente. Procedeu-se à soma da pontuação obtida na tarefa de reconto e nas respostas às questões referentes ao texto lido.

### Procedimento de análise estatística

Para discriminar os bons e maus compreendedores foi feita uma pergunta simples às professoras, que buscava inferir se a criança em questão tinha ou não problemas de leitura e/ou compreensão de texto: “Com base no desempenho do aluno X em fluência de leitura e compreensão leitora, você o classificaria como bom ou mau leitor?”. A partir da categorização feita pelas professoras, em uma dessas duas possibilidades, isto é, bons ou maus leitores/compreendedores, foram construídas curvas ROC (*Receiver Operating Characteristics curve*) e calculadas as áreas abaixo delas. Usar o professor como *gold-standard* é um procedimento comum e usualmente feito em pesquisas em diversas áreas do desenvolvimento infantil^(11,12)^.

Esse procedimento foi utilizado com as duas medidas de desfecho do estudo: taxa e acurácia. A medida contínua que demonstrasse os melhores resultados seria utilizada para estimar os prováveis pontos de corte para as crianças do 2º ano de escolarização. Caso as medidas não diferissem, a mais facilmente coletada (a saber, a taxa) seria utilizada. A estimativa dos valores de ponto de corte para o 2º ano foi feita a partir da progressão dos pontos de cortes das crianças dos demais anos escolares por meio do programa *DataFit* 9 (https://datafit.software.informer.com/9.0/). Assim, os três valores de ponto de corte obtidos, um para cada ano escolar, foram plotados e, a partir deles, diferentes possibilidades de trajetórias de crescimento foram testadas frente a um acréscimo de um novo ano escolar (i.e., o 2º ano). Sabendo-se que a distância entre os quatro anos no que tange o eixo x é equidistante, foi possível estimar, para cada modelo preditivo avaliado, possibilidades para o valor referente ao eixo y (taxa) para 2º ano. Deu-se preferência por trajetórias não lineares, dada a própria conformidade dos três pontos iniciais.

## RESULTADOS

A Tabela 1 mostra a área abaixo da curva ROC para a taxa e a acurácia, seus erros-padrão e significância estatística, a partir de uma pressuposição não erros-padrão paramétrica, para os três anos. A partir dessas áreas, verificou-se que ambas as medidas apresentaram valores maiores que 0,80. Há uma progressão não-linear dos valores de ponto de corte em relação ao desenvolvimento da criança.

**Tabela 1:** Área sob a Curva ROC para os três anos escolares e valores de ponto de corte, sensibilidade e especificidade para as medidas de taxa e acurácia na leitura

**Ano**	**Medidas**	**Área sob a curva ROC**	**S. E.**	**Corte**	**Sensibilidade**	**1-especificidade**
**3º**	**Taxa**	0,970	0,027	65,670	0,900	0,100
	**Acurácia**	0,970	0,029	58,900	0,900	0,050
**4º**	**Taxa**	0,866	0,055	70,930	0,857	0,132
	**Acurácia**	0,888	0,049	63,770	0,905	0,132
**5º**	**Taxa**	0,849	0,051	91,375	0,900	0,289
	**Acurácia**	0,836	0,052	81,575	0,850	0,263

**Legenda:** Para todas as variáveis, p < 0,001; S. E. = *standard* erro; **3º ano:** Camargo^(13)^; **4º ano:** Lobato^(14)^; **5º ano:** Vasconcelos^(15)^.

Estatisticamente, os valores da área sob a curva ROC não diferiram para taxa ou para acurácia (i.e., produziram em todos os anos áreas, sob a curva, semelhantes). Pela facilidade de coleta da taxa, utilizou-se esta medida para a estimação. Assim, a estimação do ponto de corte do 2º ano baseou-se em quatro funções não lineares: exponencial, polinomial de primeira ordem, logaritmo modificado e exponencial modificado^(16)^. Uma vez que os resultados obtidos na curva ROC, para cada um dos três anos (dados reais), não se encaixaram perfeitamente para cada uma das funções não-lineares testadas, conduziram-se aproximações. Originalmente, os valores obtidos para 3º, 4º e 5º ano estão descritos na Tabela 1; porém, a Figura 1, mostra que os valores originais sofrem aproximações para se adequarem em cada um dos quatro modelos não-lineares usados para estimar o valor da taxa do 2º ano. A Figura 1 mostra esses valores de aproximações e, para cada um dos dados reais, há uma função e uma estimação do ponto de corte específica para o 2º ano. Uma média dos três pontos de corte estimados para o 2º ano foi computada e essa média refere-se ao ponto de corte final adotado para esse ano (ou seja, (53,08 + 50,40 + 43,35 + 41,24)/4= 47,01.

**Figura 1:** Pontos de corte por ano a partir dos quatro modelos de estimação

## DISCUSSÃO

Procedimentos de rastreamento são de rápida aplicação e são utilizados para o necessário monitoramento do desenvolvimento da leitura durante a escolarização^(1,5,8)^. O acompanhamento das capacidades leitoras pode ter como objetivo não somente o conhecimento do nível de leitura do escolar, como também a identificação precoce de dificuldades de leitura de forma a prover informações para a intervenção e o suporte educacional ou clínico^(1)^. Geralmente acompanham programas de RTI e fornecem o suporte para o acompanhamento do escolar.

Conhecer valores liminares de parâmetros de fluência leitora pode ser útil para compor amostras de estudantes em pesquisas sobre a compreensão de leitura, uma vez que ambas as competências mostram associação em diferentes anos escolares^(3,4)^. Este estudo calculou e apresentou pontos de cortes para rastreamento por taxa ou acurácia mínimas para a realização de leitura com compreensão para os anos iniciais do ensino fundamental^(2,6)^. Por não haver diferenças aparentes na área sob a curva ROC entre a taxa e a acurácia de leitura, optou-se por usar uma medida que pode ser usada facilmente e sem cálculo, a saber, a taxa. Assim, os números 65, 70 e 91 de p.p.m. foram considerados valores mínimos de fluência leitora como condição para garantir a compreensão de leitura no 3º, 4º e 5º ano do ensino fundamental, respectivamente. Os textos utilizados (Material Suplementar) constituíram-se em ferramenta útil, breve e fácil para rastrear a velocidade com vistas à compreensão.

Os valores obtidos para a taxa e acurácia mostraram uma queda na especificidade (i.e., probabilidade de detecção de verdadeiro positivo) do 3º para o 5º ano (Tabela 1). É possível que os professores sejam menos sensíveis às dificuldades de compreensão de crianças maiores. Por exemplo, em estudo com escolares brasileiros^(17)^ foram encontrados sete casos de leitura deficiente de palavras reais e 12 de decodificação entre crianças de 10 anos julgadas competentes pelas professoras. É possível também que nesse grupo de crianças, por terem a fluência leitora mais desenvolvida, outros fatores, como vocabulário e capacidade de monitoramento, tenham interferido mais na compreensão leitora.

Uma das limitações deste estudo é que, embora tenham mostrado diferenças significantes em seus desempenhos, os grupos experimentais foram formados por opinião dos professores sobre a presença de dificuldades genéricas de leitura ou específicas de interpretação e compreensão leitora^(7,10)^. Relatos e indicações dos professores são vistos como importante fonte primária de informação para os demais componentes da equipe educacional^(12,16)^ e, por isso, têm papel fundamental no acompanhamento das diferentes etapas do aprendizado. Entretanto, outros estudos devem ser conduzidos com grupos clínicos, com diagnóstico de problemas específicos de aprendizagem, para melhorar as informações trazidas pela área sob a curva ROC, principalmente nos anos finais em que os valores de sensibilidade e especificidade foram menos adequados, embora ainda satisfatórios (Tabela 1).

Espera-se que esses valores sejam parecidos, caso outros textos narrativos adequados ao ano escolar e com mesmas características linguísticas dos aplicados neste estudo, sejam utilizados em procedimento semelhante. Assim, pesquisas que possibilitem saber se outros textos produziriam taxas similares aos pontos de corte obtidos, ainda devem ser realizadas. Sabe-se que textos variam conforme o tipo (narrativo ou expositivo), frequência de palavras, nível de conhecimento necessário para o entendimento, e outros aspectos que interferem em sua complexidade. Por isso, recomenda-se fortemente, que os mesmos textos narrativos usados neste estudo sejam utilizados para a identificação do valor mínimo de palavras lidas por minuto para garantir a compreensão do texto lido. Essa recomendação visa, inclusive, à redução de possível margem de erro.

Outra limitação importante é o fato de os pontos de corte das crianças mais jovens terem sido estimados (Figura 1). Apesar disso, é necessário reconhecer que valores estimados de pontos de corte produzem resultados fidedignos em situações em que os erros de medida são aceitáveis, como o caso dos obtidos na presente pesquisa^(18)^.

Ainda assim, ressalta-se que o valor aqui estimado é mais apropriado e mais bem destinado a pesquisas científicas, que a procedimentos de rastreamento clínico ou educacional. Essa limitação foi superada, em certa medida, pela estimação dos pontos de corte por meio da estatística aqui utilizada. Dessa forma, os clínicos e pesquisadores podem utilizar um texto apropriado para crianças de 2º ano com a expectativa de desempenho para os pontos de corte adotados.

Pesquisadores e clínicos poderão se beneficiar desses dados para avaliação rápida e em futuras pesquisas com amostra ampliada de crianças na faixa etária estudada e que forneçam validação cruzada dos valores aqui apresentados. Outra possibilidade é investigar se os pontos de corte aqui fornecidos são válidos para separar crianças escolares em bons e maus compreendedores, também, em amostras independentes, por meio de validação cruzada.

## CONCLUSÃO

Estabeleceram-se pontos de corte para taxa e acurácia de leitura oral de texto para escolares do 2º ao 5º ano, com recomendações de utilização dos valores da taxa de leitura de cada ano escolar para procedimentos de rastreamento da compreensão leitora.

## Material Suplementar

Este artigo acompanha material suplementar.

Textos selecionados para a pesquisa, extraídos de livros de Língua Portuguesa utilizados nas escolas, e questões correspondentes

Este material está disponível como parte da versão online do artigo na página: https://doi.org/10.1590/2317-1782/20232021263pt
